# esMPRA: an easy-to-use systematic pipeline for MPRA experiment quality control and data analysis

**DOI:** 10.1093/bioinformatics/btaf315

**Published:** 2025-05-21

**Authors:** Jiaqi Li, Pengcheng Zhang, Xi Xi, Xiaowo Wang

**Affiliations:** Ministry of Education Key Laboratory of Bioinformatics; Center for Synthetic and Systems Biology; Bioinformatics Division, Beijing National Research Center for Information Science and Technology; Department of Automation, Tsinghua University, Beijing 100084, China; Ministry of Education Key Laboratory of Bioinformatics; Center for Synthetic and Systems Biology; Bioinformatics Division, Beijing National Research Center for Information Science and Technology; Department of Automation, Tsinghua University, Beijing 100084, China; Ministry of Education Key Laboratory of Bioinformatics; Center for Synthetic and Systems Biology; Bioinformatics Division, Beijing National Research Center for Information Science and Technology; Department of Automation, Tsinghua University, Beijing 100084, China; Ministry of Education Key Laboratory of Bioinformatics; Center for Synthetic and Systems Biology; Bioinformatics Division, Beijing National Research Center for Information Science and Technology; Department of Automation, Tsinghua University, Beijing 100084, China; College of Artificial Intelligence, Tsinghua University, Beijing 100084, China

## Abstract

**Motivation:**

Massively Parallel Reporter Assays (MPRAs) have emerged as pivotal tools for systematically profiling cis-regulatory element activity, playing critical roles in deciphering gene regulation mechanisms and synthetic regulatory element engineering. However, MPRA experiments involve multi-step library processing procedures coupled with high-throughput sequencing. Operational errors during these complex workflows can lead to substantial resource depletion and experimental delays. Thus robust and user-friendly quality control methods are essential to minimize experimental failures and ensure reproducibility between replicates.

**Results:**

Here, we present esMPRA, an integrated quality control and analysis pipeline designed for MPRA experiments. Building on our experience in MPRA and its derivative techniques, coupled with systematic analysis of public MPRA datasets, we established standardized quality control metrics and developed a stepwise quality monitoring framework. esMPRA generates stage-specific diagnostic reports and provides experimental recommendations to avoid potential risks throughout the workflow. Designed for maximal accessibility, esMPRA features a one-line command-line interface and requires minimal bioinformatics expertise. Beyond quality assessment, the pipeline delivers processed data outputs, comprehensive analysis reports, and interface files compatible with downstream analyses, establishing an end-to-end solution for MPRA experimentation.

**Availability and implementation:**

esMPRA is released as an open-source software under the MIT license. The source code for esMPRA is available on Zenodo (DOI: 10.5281/zenodo.15362711) and GitHub (https://github.com/WangLabTHU/esMPRA/) for Linux, macOS, and Windows and is available via PyPI as esMPRA. Data for testing and reference is available via Zenodo repository at https://zenodo.org/records/15034449.

## 1 Introduction

Gene regulation constitutes the fundamental mechanism underlying biological processes, with cis-regulatory elements (CRE) serving as critical components that realize precise transcriptional control. Accurately quantifying the functional activity of these regulatory elements holds significant implications for deciphering the genetic code (Sharon *et al.* 2012), advancing precision medicine platforms (Huang *et al.* 2019), and optimizing bioengineering applications (Hossain *et al.* 2020). The Massively Parallel Reporter Assay (MPRA) has emerged as a pivotal technological innovation, enabling high-throughput functional characterization of cis-regulatory elements. This powerful approach has been widely adopted in diverse researches including non-coding variant analysis (Tewhey *et al.* 2016), synthetic regulatory element design (Gosai *et al.* 2024, Li *et al.* 2025), and transcriptional regulation studies (Duttke *et al.* 2024).

However, our investigations reveal substantial challenges in MPRA implementation. The technical complexity of MPRA workflows imposes significant demands on experimental operators, particularly for novice researchers at this technology, who often require extensive trial-and-error optimization that consumes substantial time and resources. Additionally, the data processing workflow for MPRA experiments is complex, often requiring researchers to expend considerable effort in handling and analyzing high-throughput data at every stage. While Tewhey *et al.* (2016) provided a companion data processing tool, MPRASuite, it lacks integrated quality control functionality and presents a steep learning curve for beginners. On the other hand, MPRAnalyze (Ashuach *et al.* 2019) enables more advanced statistical analyses of MPRA data, but it does not support the processing of raw sequencing data. The lack of easy-to-use and efficient MPRA data processing tools leads to low efficiency in the workflow of non-expert researchers.

To address these challenges, we developed esMPRA, a user-friendly computational pipeline for systematic quality control and analysis of MPRA data. Building on extensive experimental experience with MPRA, we identified key metrics at each step of the experimental workflow. Leveraging a large collection of MPRA datasets that support the CODA platform (Gosai *et al.* 2024), we generated a comprehensive set of reference samples. Based on these references, we established a quantitative quality control framework to guide and standardize MPRA data assessment. The pipeline automatically generates stepwise QC reports that identify risk factors and provide actionable troubleshooting recommendations, effectively minimizing experimental failures and reducing optimization cycles. Notably, esMPRA is exceptionally accessible to novices, with installation and operation achievable via a one command-line instruction, while delivering comprehensive analysis outputs including processed data and standardized file formats for downstream analyses (Ashuach *et al.* 2019). This integrated framework significantly enhances the efficiency and reliability of MPRA experiments while lowering technical barriers for new users.

## 2 Overview

**Figure 1.**
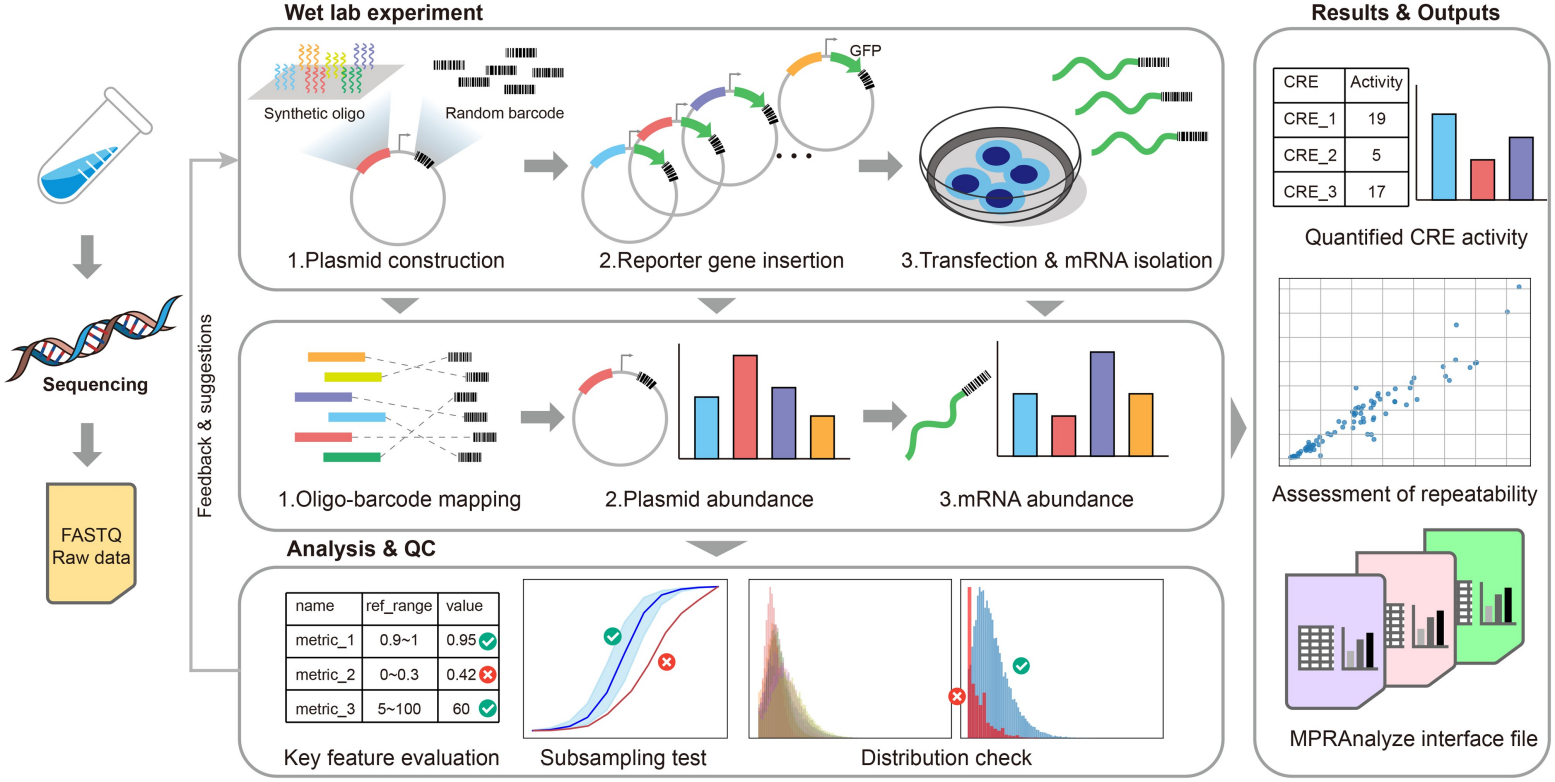
Workflow of esMPRA. esMPRA takes the raw fastq files generated in each experimental step as input and produces corresponding analytical results. While conducting the analysis, esMPRA performs quality control on key parameters in each step, providing reference ranges based on a large amount of existing MPRA data analysis and offering specific experimental suggestions for high-risk items. After completing the quality control and analysis for each step, esMPRA provides the final analytical results, assesses the reproducibility of the experiment, and outputs interface files in a standardized format for further in-depth analysis.

esMPRA is developed in Python (version 3.8.0, compatible with versions 3.7 and above) and is designed based on the experimental framework described by (Tewhey *et al.* 2016). esMPRA can run on operating systems that support Python. It can operate on a wide range of devices, from high-performance computing devices to lightweight laptops, as long as the device has sufficient RAM to handle high-throughput sequencing data. It provides dedicated data processing and quality control modules for each experimental step (Fig. 1). After organizing and integrating the codebase, esMPRA can be installed with a single pip install command, and all of its functionalities can be executed via one-line command executions. For MPRA experiments following the protocol outlined by (Tewhey *et al.* 2016), the default parameters of esMPRA are sufficient to perform all necessary functions. For each quality control step, esMPRA generates a detailed report that highlights potential risk factors and recommends appropriate experimental interventions. All parameters are comprehensively documented within the embedded help messages and the README file, allowing users to easily adjust settings as needed. Users are encouraged to consult the README for detailed step-by-step instructions and can also download curated test datasets to verify the operation of the example scripts. A script that includes all processing steps is also provided. Executing this script can automatically perform all the processing steps for the test datasets, thereby facilitating a better understanding of the overall workflow for readers.

## 3 Design and implementations

### 3.1 Quantitative quality control

Based on our previous MPRA experiments and data processing experience, we have summarized key quantitative quality control metrics for each step of the workflow. The most important of these metrics fall into the following categories: the proportion of qualified oligos (used to determine whether a sufficient number of oligos are available at each step based on specific qualification criteria), the number of barcodes associated with each oligo (used to assess the complexity of library construction and sequencing quality), the proportion of barcodes detected a specific number of times in sequencing and the mean detection count of barcodes (used to evaluate whether the number of different barcodes is evenly distributed within the sample and whether the sequencing depth is sufficient.), and the correlation between plasmid abundance and RNA abundance (used to assess whether plasmid DNA has been adequately removed). Additional detailed parameters are also included and are described in the QC reports. We calculated these metrics across a large collection of MPRA datasets (Gosai *et al.* 2024) and used the resulting values to define reference ranges. When a single step contains risk warning, users are advised to inspect the experimental procedures to address potential issues.

In addition, we simulated the effect of different sequencing depths on sequence coverage through downsampling and visualized the results to help determine whether the current sequencing depth is sufficient. And we plotted the distribution of barcode counts to assess library complexity and sequencing quality. We also established reference plots based on the collection of MPRA datasets. Furthermore, each report file includes additional analysis parameters and figures at the end to provide readers with a more comprehensive view of the experimental quality.

### 3.2 Mapping oligos with barcodes

MPRA quantifies regulatory activity by appending random barcodes to designed oligo sequences, thereby labeling a vast array of oligos for activity measurement. In addition, it aggregates the activity of the same oligo across multiple barcodes to mitigate experimental variability. In esMPRA, the “step1_oligo_barcode_map” module determines the correspondence between oligos and their random barcodes by processing the raw fastq files generated in this step. Two operational modes are available for this process; the recommended approach is to employ the—use_flash option after installing the FLASH2 tool (Magoč and Salzberg 2011), which enhances both processing speed and accuracy.

In this step, a critical metric is the number of unique random barcode types associated with each oligo sequence. It is essential to control the library complexity within an optimal range to ensure the experiment's success. An excessive number of barcodes indicates an overly complex library, which may result in many barcodes going undetected in subsequent experiments. Conversely, a barcode count that is too low can introduce significant noise during the quantification of CRE activity. For additional key metrics and further details, please refer to the README documentation.

### 3.3 Determine plasmid abundance

After mapping the correspondence between oligos and random barcodes, typically a reporter gene is inserted between them to enable the assessment of transcriptional regulatory element activity. For accurate quantification of these elements, the relative abundance of plasmids serves as a reference. Consequently, following the insertion of the reporter gene, sequencing is performed to quantify the relative abundance of each random barcode across all plasmids. The “step2_get_plasmid_counts” module accomplishes this by processing the raw fastq files generated at this stage.

A key metric in this step is the sequencing coverage of both barcodes and oligos. It is crucial that as many barcodes and oligos as possible are adequately covered by sequencing. Insufficient coverage may suggest low efficiency in plasmid construction or an inadequate sequencing depth.

### 3.4 Getting RNA (cDNA) abundance

After the insertion of the reporter gene and the quantification of barcode abundance in the plasmids, the constructed plasmids are transfected into target cells. After a defined period of culture, the RNA transcripts are measured. Typically, RNA quantification is performed by reverse transcription followed by cDNA sequencing, which indirectly determines RNA abundance. The “step3_get_RNA_counts” module processes the raw fastq files generated during this step to quantify the relative abundance of random barcodes within the cDNA, thereby enabling the accurate quantification of transcriptional regulatory element activity.

In this step, the sequencing coverage of barcodes and oligos remains a critical parameter. However, assuming that the previous quality control standards have been met, inadequate coverage in this phase may be due to various factors. The most likely cause of risk values here is an insufficient RNA input for reverse transcription, which should be checked with the provided reports.

### 3.5 Quantifying the activity

Mapping oligos with barcodes establishes the correspondence between each oligo and its random barcode. By integrating the relative abundance of barcodes measured in plasmids with that in RNA (cDNA), the regulatory activity associated with each barcode can be quantified. With the established oligo–barcode correspondence, the transcriptional regulatory activity of each oligo sequence can be evaluated, as defined by Equation (1).


(1) $$\mathrm{CRE}\;\mathrm{activity}=\frac{\sum \left(\mathrm{Normalized}\;\mathrm{RNA}\;\mathrm{counts}\;\mathrm{for}\;\mathrm{all}\;\mathrm{barcodes}\right)}{\sum \left(\mathrm{Normalized}\;\mathrm{plasmid}\;\mathrm{counts}\;\mathrm{for}\;\mathrm{all}\;\mathrm{barcodes}\right)}$$

The “step4_get_result” module quantifies the final CRE activity based on the results of these steps.

This step focuses on a single key parameter: the correlation coefficient between plasmid and cDNA abundances, which can provide significant quality control insights. An excessively high correlation suggests that DNA was not effectively removed prior to reverse transcription, potentially influencing the final quantification results. Special monitoring of this parameter is recommended.

### 3.6 Extended functionality

After completing the standard data processing and quality control steps described above, the CRE activity of the oligos obtained in a single experiment can be determined. In typical applications, several parallel replicates are conducted to ensure experimental reproducibility. To support this common requirement, esMPRA provides the “step5_compare_diff_rep” function, which evaluates the pairwise correlations among multiple replicate experiments.

In addition to the aforementioned data analysis and quality control, users may sometimes require further data processing and analysis using other tools. To facilitate such workflows, esMPRA generates standardized output files for downstream analysis. Specifically, the “generate_data_for_MPRAnalyse” function produces interface files formatted according to the requirements of MPRAnalyze (Ashuach *et al.* 2019).

## 4 Conclusion

We propose esMPRA with the aim of simplifying MPRA experiment quality control and data analysis for a broad range of users, thereby facilitating the wider adoption of this important technology. esMPRA is designed for one-line installation and operation across diverse computing platforms, drastically reducing the barrier to entry—allowing even users with minimal command line experience to get started quickly. The tool generates comprehensive quality control reports and provides experimental recommendations that help researchers rapidly identify potential risks, significantly enhancing experimental efficiency. Furthermore, esMPRA can directly produce analytical results and offers a standardized interface. The flexible and versatile framework of esMPRA also makes it applicable to a wide range of studies involving the quantification realized by random barcodes.
